# Extracapsular extension of pN2 lymph node metastases is not prognostically significant in surgically resected patients with non-small cell lung cancer

**DOI:** 10.1515/iss-2022-0023

**Published:** 2023-05-04

**Authors:** Christin Müller, Samantha Taber, Joachim Pfannschmidt, Sergej Griff

**Affiliations:** Department of Tissue Diagnostics, HELIOS Klinikum Emil von Behring, Berlin, Germany; Department of Thoracic Surgery, Heckeshorn Lung Clinic – HELIOS Klinikum Emil von Behring, Berlin, Germany; Brandenburg Medical High School Theodor Fontane Neuruppin, Neuruppin, Germany

**Keywords:** extra capsular extension, N-descriptor, non-small cell lung cancer, prognosis, staging

## Abstract

**Objectives:**

In patients with non-small cell lung cancer (NSCLC) the pathologic lymph node status N2 is a heterogeneous entity, with different degrees of lymph node involvement representing different prognoses. It is speculated whether extra capsular nodal extension may help to define a subgroup with implications on long-term survival.

**Methods:**

We retrospectively identified 118 patients with non-small cell lung cancer (65 men, 53 women), who were treated between 2013 and 2018 and found to have pathologic N2 lymph node involvement. In all patients lung resection with systematic mediastinal and hilar lymph node dissection was performed with curative intent. In N2 lymph node metastases capsules of affected lymph nodes were examined microscopically as to whether extracapsular extension was present.

**Results:**

51 patients (43 %) had extracapsular extension (ENE). Most of these patients (n=35) only had ENE in a single lymph node (69 %). The overall 5-year survival rate was 24.6 % and progression-free survival rate 17.8 %. In the multivariate analysis OS was worse for patients with multiple affected pN2 stations, concurrent N1 metastases, increasing age, and larger tumor size. For the percentage of lymph nodes affected with ENE (of total examined) only a non-significant trend towards worse OS could be observed (p=0.06).

**Conclusions:**

Although we could not demonstrate significant prognostic differences between N2 extra capsular nodal involvement within our patient population, other analyses may yield different results. However, clinicians should continue performing thorough lymph nodes dissections in order to achieve local complete resection even in patients with extra capsular tumor spread

## Introduction

Despite at least 30 years of increasingly aggressive anti-smoking campaigns, as well as advances in early recognition and targeted immunotherapies, non-small cell lung cancer (NSCLC) remains a leading cause of death in the Western world [1, 2]. If discovered early enough, surgery remains a mainstay of treatment [3, 4]. The extent to which adjuvant chemotherapy and radiation should play a role is largely determined by the tumor stage at time of surgery but remains controversial. For patients with pN2 status, according to the TNM staging system, adjuvant treatment is almost universally recommended. And yet pN2 represents a heterogenous group. Although a more precise differentiation of the N indicator has been discussed [5], none of the characteristics considered were incorporated into the most recent 8th edition of the TNM staging system [1]. As it stands, a single, enclosed metastasis to a mediastinal lymph node assigns the patient to the pN2 category. The number of nodes and stations affected is not considered. The morphology of the affected lymph nodes does not play a role in staging either. The presence of extra capsular extension of lymph node metastases (ENE) is considered in the TNM staging system for some types of cancer: in head and neck cancer, for example, ENE (defined as N3b) is suggested to have a significant detrimental effect on prognosis [6]. Although it does not currently affect staging in NSCLC, there is some evidence that ENE may play a significant prognostic role in NSCLC as well [7], [8], [9]. In this monocentric investigation of surgically resected NSCLC patients with pN2 metastases, we seek to determine, whether ENE had a significant negative impact on prognosis.

## Patients and methods

**Figure 1:**
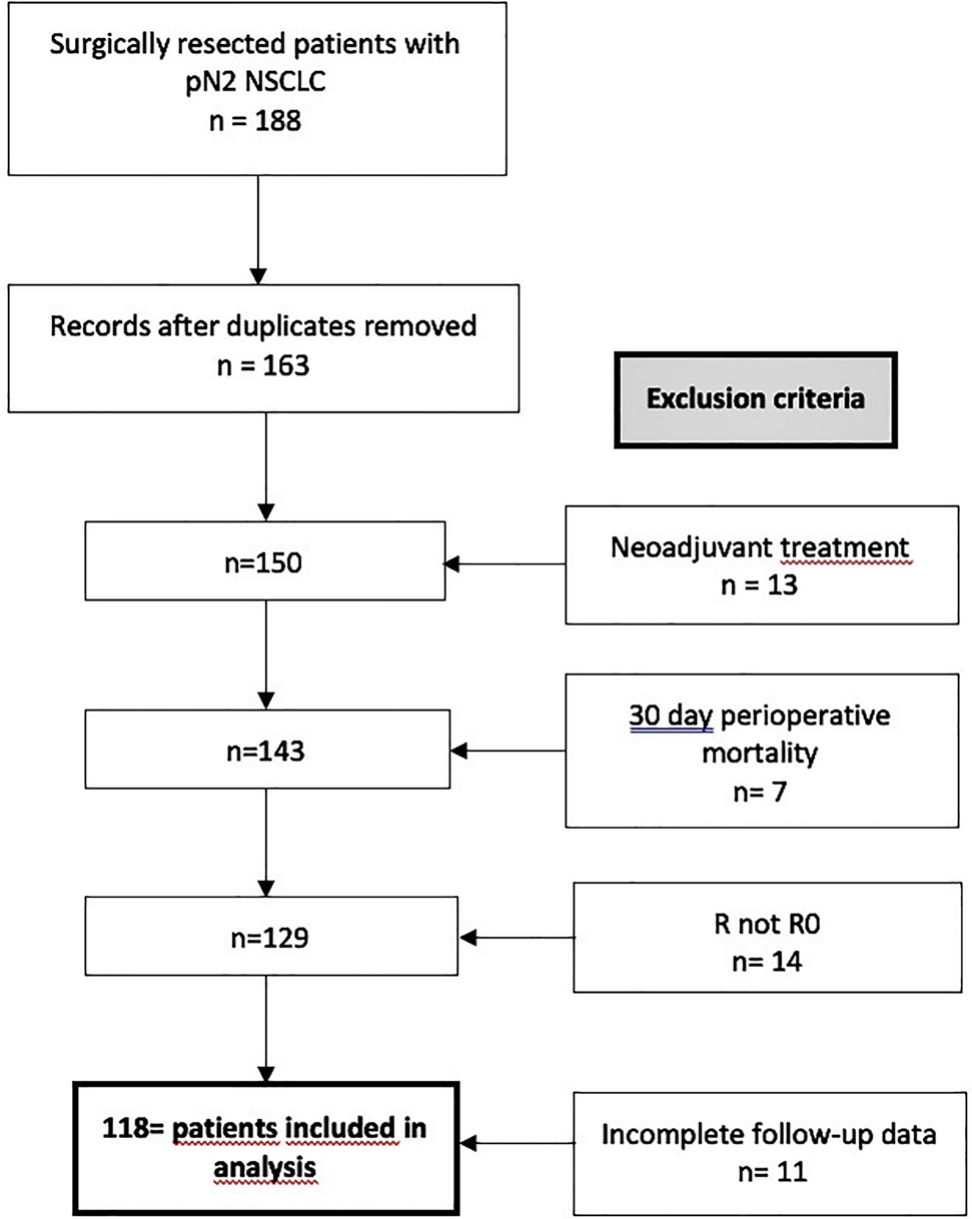
Patients included in analysis.

Our study included 118 patients, who received curatively intended surgical resection for NSCLC between January 2013 and December 2017. Only patients, who had received complete lymph node dissection and had histologically established metastases of the mediastinal lymph nodes were included. Patients with neoadjuvant treatment, non R0 resections, or death within 30 days of surgery were excluded. Patients with recurrent tumors were also excluded. Patient selection is represented visually in Figure 1.

Preoperative staging included physical examination, PET-CT of the entire body. Cerebral imaging was only performed preoperatively in cases of clinically suspected metastases.

Surgeries were usually lobectomy or extended lobectomy and, less frequently, pneumonectomy or segment resection. Systematic lymph node dissection [10] included complete removal of hilar, interlobar, lobar, intralobar, and mediastinal lymph nodes – when possible, as complete compartments. As is recommended practice mediastinal nodal resection on the right side included lymph node positions 7, 8, 9, 2, and 4; left sided resection included lymph node positions 7, 8, 9, 5, and 6.

### Microscopic examination

**Figure 2:**
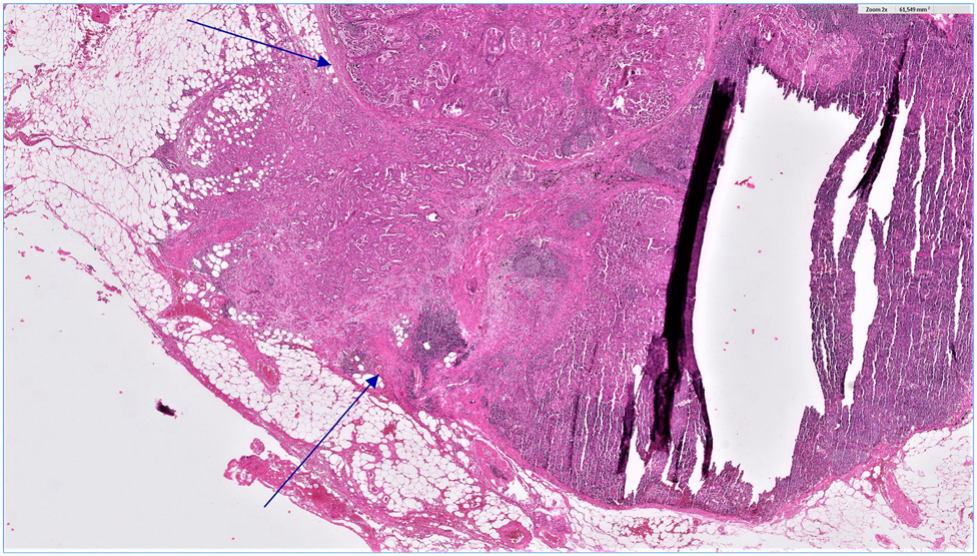
Lymph node with extracapsular extension.

All resected lymph nodes were retrospectively examined by board certified pathologists, and patients with one or more metastasis in an N2 position were included in this analysis. The presence or absence of N1 metastases was also documented. All lymph node and tumor specimens were fixed in formalin, sectioned into 3–5 mm slices, stained with hematoxylin-eosin and examined microscopically. After malignancy was established, further characteristics were determined: histological type, differentiation grade (G), tumor size (largest diameter), and when possible vascular or lymphatic invasion. Finally, the capsules of affected lymph nodes were examined microscopically by two board-certified pathologists, and a consensus was reached as to whether ENE was present. We defined ENE of nodal metastasis as continuous extension of metastatic cells through the nodal capsule into the perinodal tissue. Fragmented lymph node tissue with metastasis at the edges but no clearly recognizable capsule was classified as non ENE. Tumor deposits found only in surrounding fat tissue were also classified as non ENE. A lymph node with extracapsular extension is presented in Figure 2.

The number and station of affected lymph nodes was documented, and the number (if >1) of lymph nodes with extracapsular extension was noted. We also noted what percentage of lymph nodes (of the number examined) were affected.

### Immunhistology and molecular pathology

All tumors were examined with immunohistochemistry for a precise entity diagnosis. Molecular analysis was performed according to standard diagnostic procedure for adenocarcinomas, sarcomatoid carcinomas and large cell carcinomas, but not for squamous carcinomas. Additional immunohistochemical analysis of lymph node metastasis was not performed.

### Follow-up

We retrospectively extracted data on clinical patient characteristics from the data bank of the Lung Clinic. These included whether or not patients had received adjuvant chemotherapy and radiation, as is recommended in cases of mediastinal nodal metastases. We also extracted data on age, gender, type of surgery, tumor histology, tumor size, pV (vascular invasion) and pL (lymphatic invasion) status, and EGFR and KRAS mutations. We also extracted data on tumor stage and reclassified it based on the currently applied 8th edition of the TNM system. Following treatment, patients were followed up with chest CT alternating with chest radiography every three months for the first two postoperative years and twice a year for the three years thereafter. In cases where patients were lost to follow-up we contacted the district resident’s registry to determine whether the patients were still alive or if they were not, when they had died. All patients had preoperatively given their informed consent to have their anonymized data and tissue specimens used for future research projects. For this reason ethics committee approval has been waived.

### Statistical analysis

Non-censored patients were followed for up 99 months, with a median follow-up time of 72 months. Overall survival (OS) time was calculated as the difference between date of surgery and date of death from any cause or last known follow-up. Progression-free survival (PFS) was the difference between date of surgery and date of established recurrence or death from any cause. The Kaplan–Maier method was used to generate estimated survival curves for OS and PFS.

The above mentioned histological and clinical characteristics were analyzed to determine their potential influences on progression-free survival (PFS) and overall survival (OS). Initial analyses were performed using the chi-squared test for categorical variables and Student’s t-test and Wilcoxon test for continuous variables. Variables where p<0.1 were considered for the multivariable analysis (Cox proportional hazards).

All statistical analyses were performed using R 3.3.0 software (R Foundation for Statistical Computing, Vienna, Austria).

## Results

### Patient characteristics

We identified 65 men and 53 women, who fit our study inclusion criteria. See Figure 1. The median age was 64.6 years. The median tumor size was 45 mm. Adenocarcinoma was the most frequently represented histology (n=83; 70.3 %), followed by squamous cell carcinoma (n=31; 26.3 %) and other forms (n=4; 3.3 %). Immunohistochemical analysis was performed in 87 patients (73.7 %). The most frequent mutations were EGFR (n=17) and KRAS (n=19). The majority of surgeries performed were lobectomies or extended lobectomies (n=99), with a much smaller number of pneumonectomies (n=18) and a single segmental resection. In 115 cases the procedure was performed as an open thoracotomy; three were performed as VATS. 114 patients (96.6 %) had adjuvant radiation, chemotherapy, or both.

### Lymph nodes

A total of 51 patients (43 %) had extracapsular extension (ENE). Most of these patients (n=35) only had ENE in a single lymph node (69 %), while 9 patients had ENE in 2 lymph nodes (18 %), and 7 patients had ENE in 3 or more nodes (14 %). In 78 of 81 ENE positive lymph nodes, the lymph node was complete. In three cases the lymph node was fragmented, but after careful examination by the pathologists it was possible to reconstruct enough to determine that the affected fragments belonged to a single lymph node so that they were not erroneously counted twice. 67 patients (57 %) were negative for ENE. Here, we counted a total of 157 lymph node specimens; 24 of these were lymph-node fragments (15 %), and the rest were complete lymph nodes. Of the 1800 lymph nodes examined, 81 lymph nodes were ENE positive. Thus, in 95.5 % of examined lymph nodes no ENE was detected. The median number of examined lymph nodes was 14, and the median percentage of affected lymph nodes was 14 %. 24 patients (29 %) had affected lymph nodes in multiple stations. 39 patients (33 %) had skip metastases where no metastases in the N1 lymph nodes were present. These data are summarized in Tables 1 and 2.

**Table 1:** Clinicopathologic characteristics of study population (categorical variable).

Variable	Number	Percent
Sex		
Male	65	(55 %)
Female	53	(45 %)
Radiation		
No	9	(7.1 %)
Yes	109	(92.9 %)
Chemotherapy		
No	8	(6.8 %)
Yes	110	(93.2 %)
Adjuvant treatment		
Yes	114	(96.6 %)
Only surgery	4	(3.4 %)
Operation		
(Bi)lobectomy	99	(83.9 %)
Segmentectomy	1	(0.8 %)
Pneumonectomy	18	(15.3 %)
Histology		
Adeoncarcinoma	83	(70.3 %)
Squamous cell	31	(26.3 %)
Other	4	(3.4 %)
T-indicator (8th Ed. TNM)		
pT1	16	(13.6 %)
pT2	43	(36.4 %)
pT3	34	(28.8 %)
pT4	25	(21.2 %)
Highest nodal station affected		
3/9	5	(4.3 %)
7/8	46	(39.7 %)
2/4 or 5/6	65	(56 %)
ENE		
Positive	51	(43 %)
Negative	67	(57 %)
ENE		
No affected nodes	67	(57 %)
One affected	35	(30 %)
≥2 affected	16	(13 %)
pN1 metastases		
Yes	79	(67 %)
No	39	(33 %)
Multiple nodal stations with metastases		
Yes	24	(20 %)
No	93	(80 %)
Lymph invasion		
L0	47	(45.6 %)
L1	56	(54.4 %)
Vascular invasion		
V0	67	(63.8 %)
V1	38	(36.2 %)
Tumor size		
≤40 mm	44	(39 %)
>40 mm	69	(61 %)
EGFR		
Positive	17	(19.5 %)
Negative	70	(80.5 %)
KRAS		
Positive	19	(21.8 %)
Negative	68	(78.2 %)

ENE, extracapsular extension.

**Table 2:** Clinicopathologic characteristics (continous variables).

Variable	
OS, months/median	38.3 (24)
PFS, months/median	29.7 (24.6)
Age, years/median	64.6 (9.6)
N2 nodes affected, %	2 (0–2)
Tumor size, mm/mean	45 (14–76)
Number ENE	0 (0–2)
Percent ENE	0 (0–7.6 %)
Number N2 nodes affected/mean	13.5 (3.5–23.5)

Data presented as mean (+/− SD) or median (IQR). OS, overall survival; PFS, disease free survival; ENE, extracapsular extension (of nodal metastases).

### Survival

**Figure 3:**
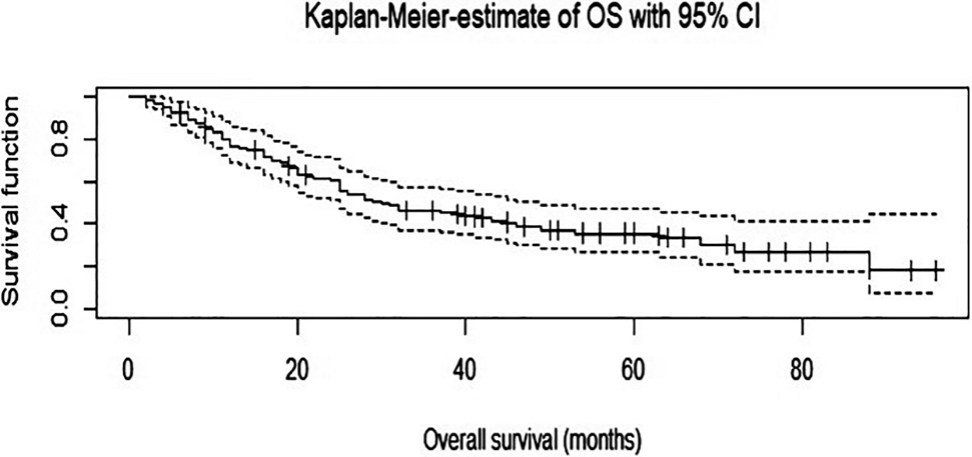
Cumulative survival curve – overall survival.

**Figure 4:**
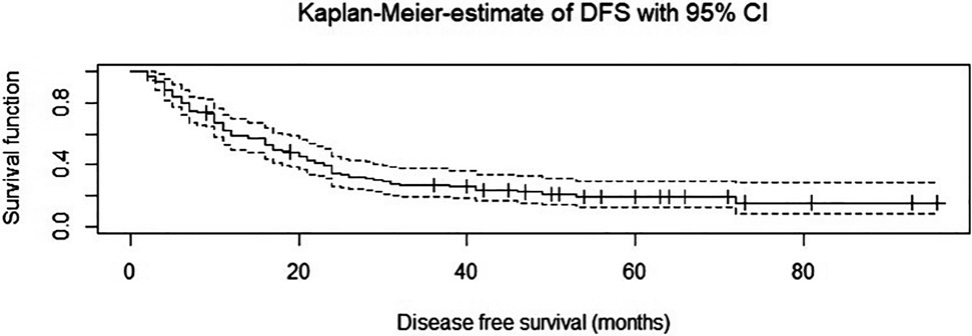
Cumulative survival curve – progression free survival (Disease free survival).

The median follow-up for non-censored patients was 72 months. The median OS was 38.3 months (SD 24), and the median PFS was 29.7 months (SD 24.6). 62 patients died during the follow-up period, and 80 patients died or had a tumor recurrence. The 5-year survival rate for OS was 24.6 %; for PFS it was 17.8 %. See Figures 3 and 4 for cumulative survival curves.

In the univariate analysis metastases in multiple lymph nodes stations, concurrent N1 metastases, no adjuvant treatment, greater age, and larger tumor size were all associated with worse OS and PFS. ENE as a binary category (y/n) was not significant, but the number and percent of lymph nodes affected with ENE was. In a post hoc stratification of ENE into 0, 1, or 2+ (number of affected LN) the effect on OS and PFS was also not significant. The total number and percent of pN2 lymph nodes (with or without ENE) was also associated with worse OS and PFS. See Tables 3 and 4. EGFR and KRAS mutations had no significant effect on OS or PFS in this collective.

**Table 3:** Univariate analysis between categorical clinicopathological characteristics and prognosis for categorical variables.

Variable	p-Value (PFS)	p-Value (OS)
Chemotherapy, y/n	0.186	0.193
EGFR, y/n	0.339	0.403
ENE, y/n	NS	NS
Highest nodal station affected	NS	NS
Histology	NS	NS
KRAS	0.568	0.44
Multiple nodal stations affected	0.004	0.004
pN1 metastases	0.045	0.08
Uncertain resection	NS	NS
Only surgery	0.022	0.0125
Operation type	NS	NS
pT status (1,2,3,4)	NS	NS
Radiation, y/n	NS	NS
Sex, m/f	0.17	0.098
Lymph invasion	NS	NS
Vascular invasion	0.089	0.2
ENE (0, 1, 2+)	NS	NS

PFS, disease free survival; OS, overall survival; ENE, extracapsular extension; NS, non-significant.

**Table 4:** Univaraite analysis for continous variables.

Variable	p-Value (PFS)	p-Value (OS)
Age	0.05	<0.001
Tumor size	<0.001	<0.001
Number ENE	<0.001	<0.001
Percent ENE	<0.001	<0.001
Total pN2 nodes	<0.001	<0.001
Percent pN2 nodes	<0.001	<0.001

PFS, disease free survival; OS, overall survival; ENE, extracapsular extension.

Since it was of interest to this study an additional category of “uncertain resection” was created post hoc. This was based on a concept developed by Gagliasso et al. [11] and includes patients with either ENE or an affected lymph node in the highest position (2/4 or 5/6). Uncertain resection, however, was not prognostically significant in our study population.

**Figure 5:**
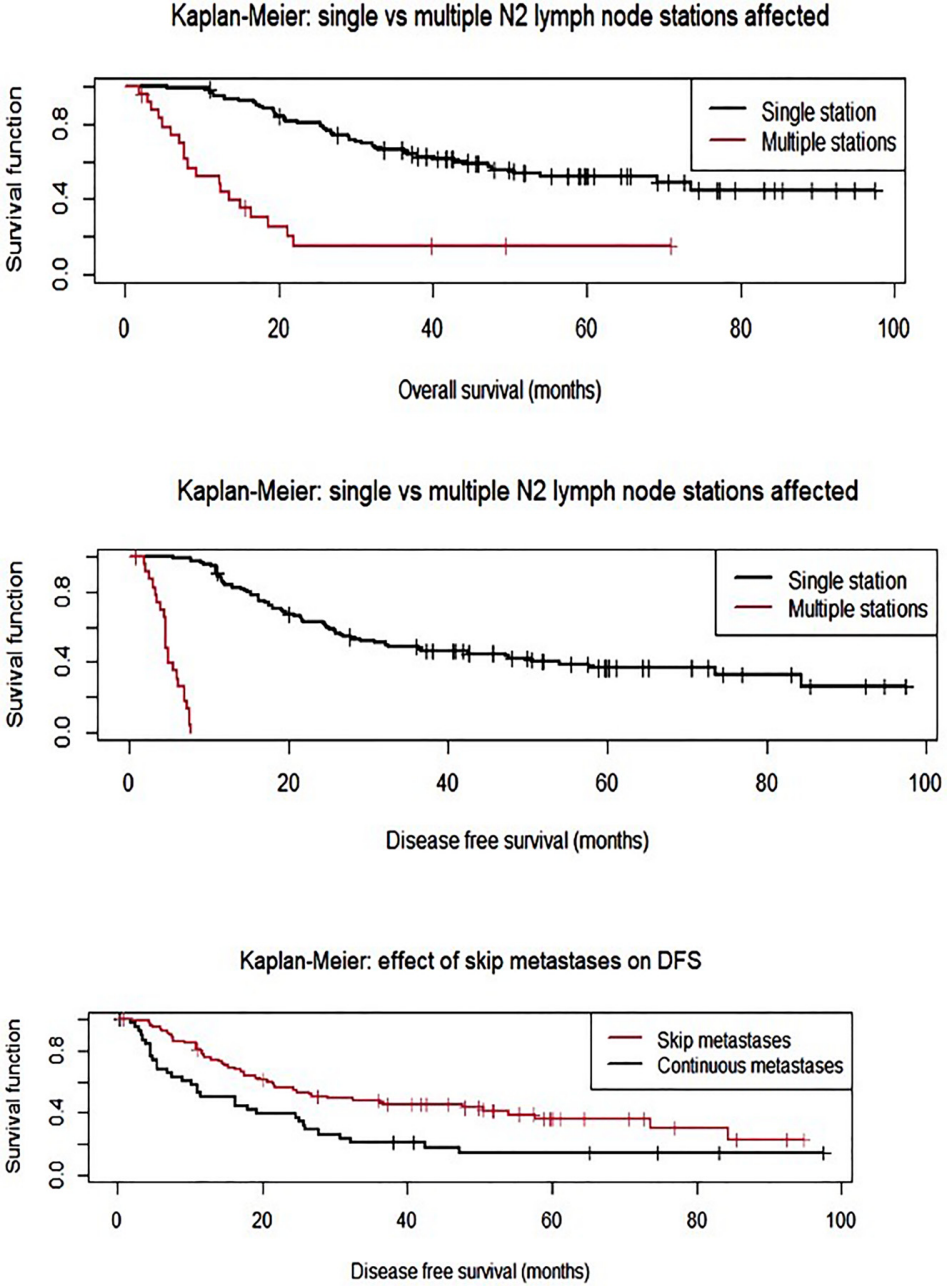
Results of multivariate analyses: The effects of concurrent N1 metastases (continuous) and multiple affected N2 nodal stations vs. single N2 nodal station on OS and PFS (DFS).

In the multivariate analysis OS was worse for patients with multiple pN2 stations, concurrent N1 metastases, increasing age, and larger tumor size. For the percentage of lymph nodes affected with ENE (of total examined) only a non-significant trend towards worse OS could be observed (p=0.06). Multiple pN2 stations and concurrent N1 metastases were associated with worse PFS as well. See Tables 5 and 6 as well as Figure 5. 26 patients (of 106 on whom data was available) had local recurrences (24.5 %). Among these patients the median time until recurrence was 22.4 months.

**Table 5:** Multivariable analysis for OS.

Variable	p-Value	Hazard ratio (95 % CI)
Multiple nodal stations affected	<0.001	8.16 (4.2–15.7)
No pN1 metastases	0.003	0.45 (0.26–0.77)
Age	0.004	1.04 (1.01–1.08)
Tumor size	0.028	1.01 (1.001–1.02)
Percent ENE	0.061	3.2 (1.1–4.2)

CI, confidence interval; OS, overall survival; ENE, extracapsular extension.

**Table 6:** Multivariable analysis for PFS.

Variable	p-Value	Hazard ratio (95 % CI)
Multiple nodal stations affected	<0.001	3.01 (1.1–4.17)
No pN1 metastases	0.007	0.51 (0.30–0.62)
Age	NS	
Tumor size	NS	
Percent ENE	NS	

CI, confidence interval; PFS, disease free survival; ENE, extracapsular extension; NS, non-significant.

We did not however find any significant relationship between ENE and likelihood of local recurrence.

## Discussion

The primary finding of this paper was that ENE did not have a significant independent impact on long-term survival or progression-free survival in surgically resected NSCLC patients with pN2 metastases. This stands in opposition to previous studies [7], [8], [9]. Although ENE was associated with worse OS and PFS in our univariate analyses, none of the measures of ENE were significant in the multivariable analyses. Here we can speculate that the more powerful factors of overall extent of nodal metastatic spread (involvement of multiple nodal stations, concurrent N1 metastases) may have outweighed any effect of ENE. These factors were not considered either in the analysis by Liu and colleagues [9] or Lee and colleagues [7]. Moreover, our study was confined to patients with pN2 metastases, whereas Liu and Lee included patients with only pN1 metastases and, as would be expected, found that these patients had better prognoses. We did find, however, that patients with concurrent pN1 metastases had worse outcomes than those with only pN2 (skip metastases). This is in concurrence with previous findings [12], [13], [14], [15]. Luchini et al. [8] found that the detrimental effects of ENE were more pronounced in cases of pN1 rather than pN2 metastases.

It is worth noting that most of our patients with ENE had only less than 3 nodes with extranodal involvement. This may have affected our results. Liu only considered presence vs. absence of ENE and did not report on the number of affected nodes [9]. Lee differentiated between 0, 1–3 and >3 nodes affected with ENE. Here, of the patients with ENE over half (53.7 %) had >3 nodes [7]. In our collective, in contrast, many patients with ENE had only a single affected node (35.7 %), whereas only three patients (5.9 %) had 3 or more nodes affected with ENE. In contrast to our study, neither of the studies mentioned above give a clear definition of ENE. For example, it is unclear whether specimens with isolated tumor cells in fat tissue and fragmented lymph node tissue were counted as ENE or not. In other studies as well, there seems to be some ambiguity as to what constitutes ENE. In a meta-analysis on the topic Luchini et al. [8] found that in some studies ENE was defined as metastatic cells appearing to extend continuously beyond lymph node capsule, whereas in others it was defined to included tumor deposits and vascular emboli observed in the surrounding adipose tissue. Theunissen et al. [16] have also pointed to some possible difficulties in achieving consistent diagnoses of extra capsular extension but argue that it is possible as long as clearly established criteria is applied. The lack of a consistent definition of ENE makes inter-study comparison difficult. Not only should surgeons and pathologists be encouraged to consider ENE in dissected lymph nodes, but for future, larger studies a consensus on ENE definition would be of utmost importance.

A secondary finding of our study is that metastases to multiple lymph nodes stations – whether ENE was present or not -- were associated with worse OS and PFS. This is in concurrence with several previous studies [17], [18], [19], [20].

It is well established that locoregional metastases are one of the most important prognostic factors in NSCLC [21, 22]. Based on the current TNM classification system (8th edition) a single, circumscribed mediastinal lymph node metastasis determines a status of pN2, usually with a consequent recommendation of sequential chemotherapy and radiation of the mediastinum. A whole range of potential considerations including morphology (i.e. “bulky”), number of affected nodes or nodal stations, vascular invasion, preoperative suspicion vs. occult metastases (cN status), tumor aggressivity (G status), or in this case the presence or absence of ENE are not incorporated into the current TNM staging system. Attempts have been made to better differentiate among pN2 cases [23], [24], [25], in particular to determine which patients will truly benefit from adjuvant therapy. Drake et al. [26], for example, determined that in patients with only microscopic N2 progression adjuvant mediastinal radiation provided no benefit. Interestingly, Moretti et al. found that adjuvant radiation only improved prognosis in pN2 patients without ENE, but not in those with ENE [27].

Although KRAS and EGFR mutations did not affect outcome in our investigation, molecular pathological examinations can be expected to play an increasingly significant role in refining adjuvant or neoadjuvant medical treatment in surgical patients. It has been well established that the prognosis of operable NSCLC is to a large extent dependent on whether or not it is truly locally contained. Postoperative examination of resection margins and lymph nodes are the main methods of estimating the chances of non-local spread. The calculation, however, is statistical; nodal metastases are associated with a higher likelihood of systemic spread but do not determine it. We wondered whether ENE status might offer a more precise tool for estimating the chances of systemic spread.

In recent years there has been some interest in liquid biopsies as a more direct method of determining systemic spread. Here, various methods exist for examining patient blood for circulating tumor cells as well as tumor cell fragments. Circulating tumor DNA (ctDNA) can be examined for relevant mutations (such as EGFR), which may aid in determining the appropriateness of immunotherapies. Liquid biopsy has the primary benefit of being virtually non-invasive, and in patients where surgery or tissue biopsy are not tenable, it may be a helpful diagnostic tool. At the moment, however, it is not considered a suitable replacement for histological examination of tissue specimens, which remains the gold standard [28].

The main limitations of this study are its retrospective nature and relatively small cohort size. Our failure to corroborate the findings others regarding the negative prognostic effects of ENE may have been a simple matter of being underpowered. Although our paper did not find that ENE had a significant effect on prognosis in NSCLC, ENE, when considered along with what appear to be other more powerful nodal characteristics, may indeed be yet one more aspect to take into consideration when making decisions about adjuvant treatment.

## Supplementary Material

Supplementary Material
